# Supplementary material for: The non-specific phospholipase C of common bean PvNPC4 modulates roots and nodule development
Source: PLoS One. 2025 May 5;20(5):e0306505. doi: 10.1371/journal.pone.0306505 (PMC12052164; doi:10.1371/journal.pone.0306505)
Supplement: Attachment — Submitted filename: Response to reviewers.docx [file pone.0306505.s007.docx]

**Review Comments to the Author**

Reviewer #1: In this manuscript, the authors reported data supporting the symbiotic role of PvNPC4 in P. vulgaris. They demonstrated that the downregulation of PvNPC4 affects root development and nodule formation. The presented data advance our knowledge about the role of NPC in the root nodule symbiosis.

**Comments:**
-Could you please elaborate on the criteria used to select PvPI-PLC4 and PvNPC4? This information is crucial for a comprehensive understanding of your research.

R. We appreciate the comment. Our criteria for selecting *PvPI-PLC4* was initially based on its transcription profile in [PvGEA (Common Bean Gene Expression Atlas (https://www.zhaolab.org/PvGEA/](PvGEA%20(Common%20Bean%20Gene%20Expression%20Atlas%20(https://www.zhaolab.org/PvGEA/)) (O’Rourke *et al.*, 2014). As observed in Fig. S7. *PvPI-PLC4* has the highest level of expression (RPKM) in roots and nodules. The PvGEA data are very useful as a reference for our studies; however, we always do qPCR of the gen of interest to corroborate the abundance of the transcripts of interest. Therefore, we performed qPCR on our samples and found barely detectable levels of the *PvPI-PLC4* transcript. For this reason, we discarded *PvPI-PLC4* for further analyses. On the other hand, *PvNPC4* is the ortholog of *A. thaliana* *NPC4*, which was previously characterized and reported that is a plasma membrane-localized protein and has a key role in degradating plasma membrane phospholipids (Nakamura et al., 2005). Since phospholipid metabolism and membrane lipid turnover are essential for nodule development, we were encouraged to study the function of *PvNPC4* in common bean-*R. tropici* symbiosis. These criteria are described in lines 325-332.
-Do the authors observe any defect in root hairs?

R. Unfortunately we were not able to analyze the root hairs microscopically due to several technical difficulties when performing that experiment. We have discussed this issue in the discussion section (lines: 640-641).

-Could defects in root development, particularly lateral roots, compromise nodule development?

-R. We appreciate the question. We consider that the mechanisms of root and nodule formation are correlated. In fact, previous studies have demonstrated the participation of some genes in the regulation of the development of both roots and nodules (Schiessl et al., 2019; Soyano et al., 2019). It is proposed that during evolution, some of the components of root developmental pathways have been recruited in nodule development (Soyano et al., 2021). In this version of the manuscript, we analyze and discuss the effect of *PvNPC4* silencing on the transcript abundance of the two *LATERAL ORGAN BOUNDARIES-DOMAIN 16 (LBD16)/ASYMMETRIC LEAVES2-LIKE 18 (ASL18)* genes involved in root development. The transcript abundance of one these genes (*PvASL18b*) was significantly reduced in *PvNPC4*-RNAi roots (see lines: 417-418 and Fig 5A). Likewise, the transcript abundance of a gene encoding an auxin transporter (*PvPin1b*) was also reduced by *PvNPC4* silencing (see lines: 419-421 and Fig 5B). Finally, we propose a model that explains the role of PvNPC4 in root and nodule development (Fig 10).
-Is it possible that negative regulators of the nodule formation (i.e., AON pathway) are active in PvNPC4-RNAi roots? It would be interesting to assess the expression of RIC and TML in PvNPC4-RNAi roots.

R. Thanks for the question. In this new version of the manuscript, we analyzed the effect of *PvNPC4* silencing on the transcript abundance of several genes involved in AON, such as *PvRIC1*, *PvRIC2*, and *PvTMLa*. The results obtained showed an increase in the abundance of the *PvRIC1* transcript, suggesting the activation of the AON pathway. See the results obtained in lines 487-496 and Fig 8A, B.

-The authors must remember that different genetic modules regulate the rhizobial infection and nodule development programs. Indeed, some mutants form several infection threads but no nodules. Please modify this in the Discussion section.

R. We agree with the observation. The discussion section has been modified as indicated throughout that section (lines 614-620 and 642-646).

-I strongly recommend that you include a clear depiction of the root phenotype as a main figure. This will greatly enhance the visual understanding of your research.

R. Thanks for the recommendation. A figure illustrating the root phenotype was included as the main figure in this version of the manuscript (Fig. 4).

**Reviewer #2**: Data on manuscript are strongly supported by a careful experimental procedure and strong statistics approaches. The data obtained supports clearly and strongly the obtained results. The in silico analysis of the PLC genes, gives a genomic supports of ancient divergence between leguminous and non leguminous plants and close relationship between bean and soybean species.
R. We really appreciate the comment. We consider that proper statistical analysis is crucial to improve data interpretation and visualization.
The paper shows evidence of the number and identity of the PLC genes family in bean and confirms the transcription pattern of root and nodule of PLC related sequences, allowing to select PLC genes involved in root and symbiotic development and function.

R. The transcriptomic data available in the repository are useful for selecting candidate genes to study in a specific process of interest, even more so when the gene family has not been reported.
The results obtained when PvNPC4 is silenced by RNA interference, demonstrated the participation of this gene in the development of primary and lateral roots. Also the knockdown of PvNPC4 reduce the infection process and the nodule development during the symbiotic interaction of bean with rhizobia.
R. We appreciate the comment. Our data reveal the role of *PvNPC4* in root and nodule development, providing further evidence for shared pathways to regulate root and nodule development, as reported (Schiessl *et al.*, 2019; Soyano *et al.*, 2019) and discussed earlier (Lebedeva *et al.*, 2021; Soyano *et al.*, 2021)
The functional analysis by PvNPC4-RNAi shows by the first-time evidence of its involvement in bean root development and the nitrogen fixing symbiosis.
R. Thank for the involvement of PLC in rhizobial symbiosis. We believe that this report can contribute to the understanding of the molecular mechanisms underlying the establishment of this symbiosis.
This results open a new area in order to further understand the molecular mechanism implicated in the symbiotic process through lipid signaling.
R. We really appreciate this comment. We are excited to further analyze and study the role of lipid-mediate signaling during rhizobial symbiosis.
Minor ortographic or typographic details:

Materials and methods:
Lotus japonicus), (Arabidopsis, (Glycine max)
transcript abundance of PvPC4 (PvPNPC4)
transcriptional profile of PvPC4 (PvPNPC4)

Fig 2. …. gene duplation (duplication) …
S10 Fig Figure title PvPC4 (PvNPC4) (Phvul.010G033400.1)

R. Thanks for the correction. These typographical errors were amended in the new version.

**Geneal comments**

Throughout the text, we have changed expressions such as: expression levels, transcript levels, and expression, for “transcript abundance”, since this is a more precise expression to refer to the results of qPCR analyses.
